# Supplementary material for: Protein acetylation affects acetate metabolism, motility and acid stress response in Escherichia coli
Source: Mol Syst Biol. 2014 Nov 28;10(11):762. doi: 10.15252/msb.20145227 (PMC4299603; doi:10.15252/msb.20145227)
Supplement: Supplementary file 1 — Supplementary Figure S1 [file msb0010-0762-sd1.pdf]

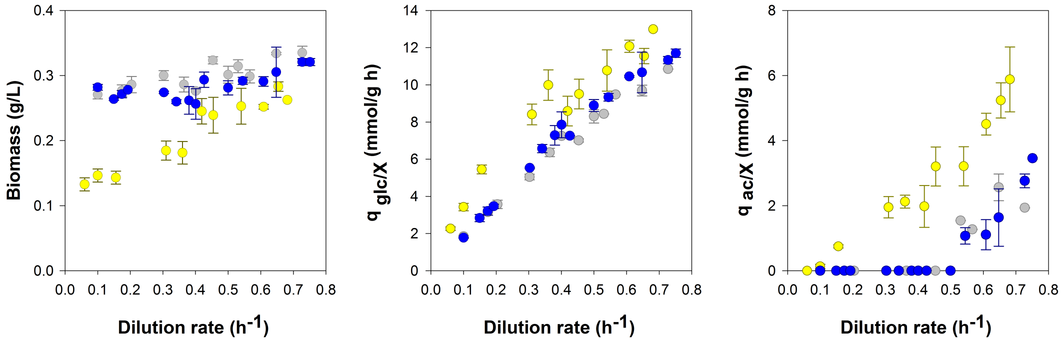

Supplementary Figure 1. *Escherichia coli* BW25113 (grey) and its knockout strains Δ*cobB* (yellow), Δ*patZ* (blue) grown in glucose-limited cultures at different dilution rates. **(A)** Cell Biomass, **(B)** glucose specific consumption rate and **(C)** acetate specific production rate of all strains as a function of dilution rates.
