# Supplementary material for: Protein acetylation affects acetate metabolism, motility and acid stress response in Escherichia coli
Source: Mol Syst Biol. 2014 Nov 28;10(11):762. doi: 10.15252/msb.20145227 (PMC4299603; doi:10.15252/msb.20145227)
Supplement: Supplementary file 2 — Supplementary Figure S2 [file msb0010-0762-sd2.pdf]

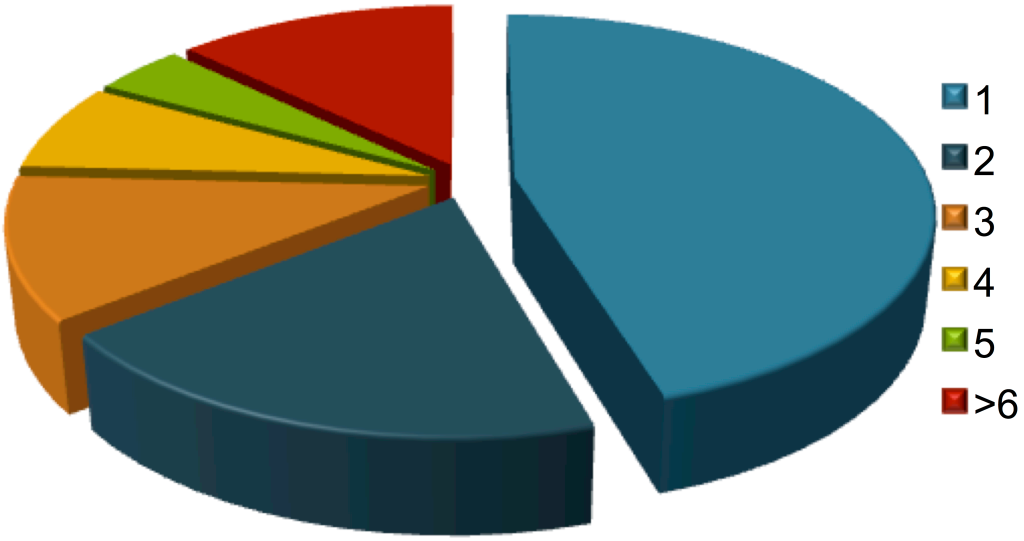

Supplementary Figure 2. Percentage of proteins with single or multiple lysine acetylation sites identified. All peptides detected in this work in all conditions assayed were considered for this analysis.
