# Supplementary material for: Protein acetylation affects acetate metabolism, motility and acid stress response in Escherichia coli
Source: Mol Syst Biol. 2014 Nov 28;10(11):762. doi: 10.15252/msb.20145227 (PMC4299603; doi:10.15252/msb.20145227)
Supplement: Supplementary file 3 — Supplementary Figure S3 [file msb0010-0762-sd3.pdf]

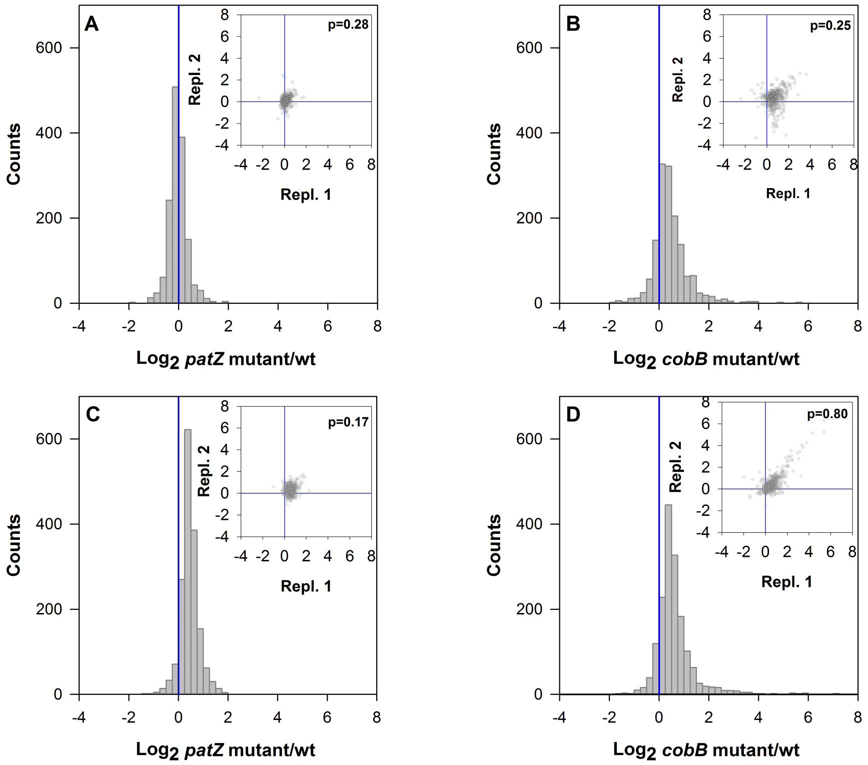

Supplementary Figure 3. Frequency histogram of the acetylated peptide ratios (Log_2) of *E. coli* Δ*patZ* and Δ*cobB* mutants referred to the wild type strain. Bacteria were grown in glucose batch cultures and bacteria were harvested prior to analysis at exponential (A, B) and stationary phase (C, D). Acetylation data are expressed as the ratios for Δ*patZ*/wt (A-C) and Δ*cobB*/wt (B-D) strains. Frequency histograms represent the median of the log_2 ratios from four biological replicates. Insert figures represent the correlation of acetylated peptide ratios in two biological replicates (pearson correlation coefficient of each condition/mutant comparison is shown on the plot).
