# Supplementary material for: Protein acetylation affects acetate metabolism, motility and acid stress response in Escherichia coli
Source: Mol Syst Biol. 2014 Nov 28;10(11):762. doi: 10.15252/msb.20145227 (PMC4299603; doi:10.15252/msb.20145227)
Supplement: Supplementary file 4 — Supplementary Figure S4 [file msb0010-0762-sd4.pdf]

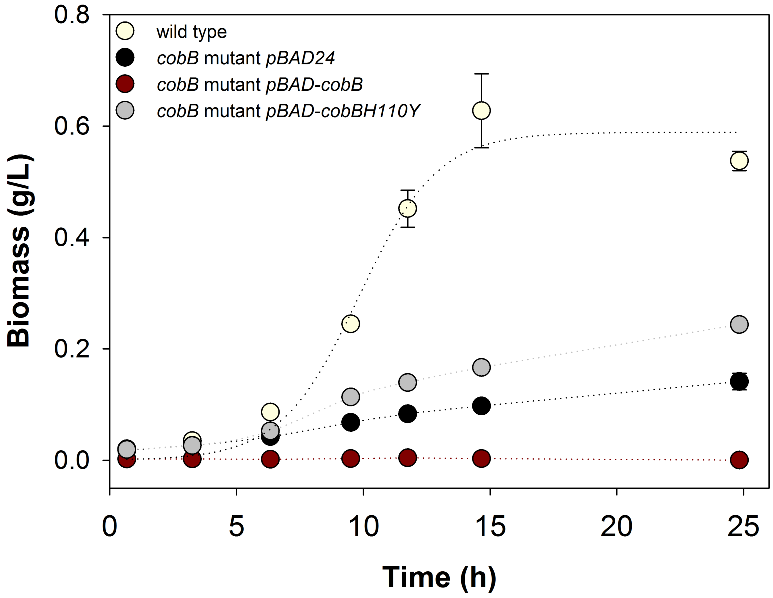

Supplementary Figure 4. *Escherichia coli* wild type (white) and its knockout mutant transformed with *pBAD24* (black circles), pBAD-*cobB* (dark red circles) and *pBAD-cobB*H110Y (grey circles) plasmids. Transformants were grown in acetate minimal medium.
