# Supplementary material for: Protein acetylation affects acetate metabolism, motility and acid stress response in Escherichia coli
Source: Mol Syst Biol. 2014 Nov 28;10(11):762. doi: 10.15252/msb.20145227 (PMC4299603; doi:10.15252/msb.20145227)
Supplement: Supplementary file 5 — Supplementary Figure S5 [file msb0010-0762-sd5.pdf]

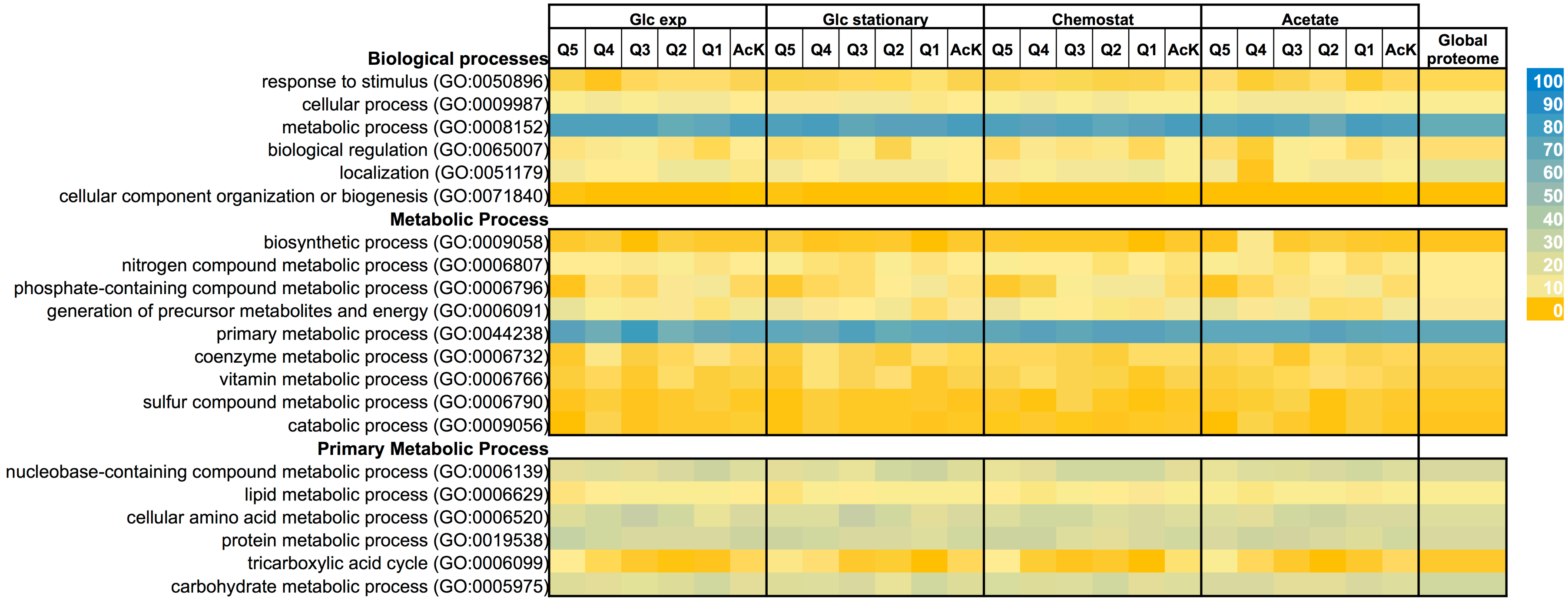

Supplementary Figure 5. Percentage of acetylated proteins in different biological and metabolic processes. The percentage is independent from protein abundance and conserved in the different condition of this study. Global proteome represents all proteins annotated in *E. coli* genome**.** Further information is detailed in Supplementary material.
