# Supplementary material for: Protein acetylation affects acetate metabolism, motility and acid stress response in Escherichia coli
Source: Mol Syst Biol. 2014 Nov 28;10(11):762. doi: 10.15252/msb.20145227 (PMC4299603; doi:10.15252/msb.20145227)
Supplement: Supplementary file 6 — Supplementary Figure S6 [file msb0010-0762-sd6.pdf]

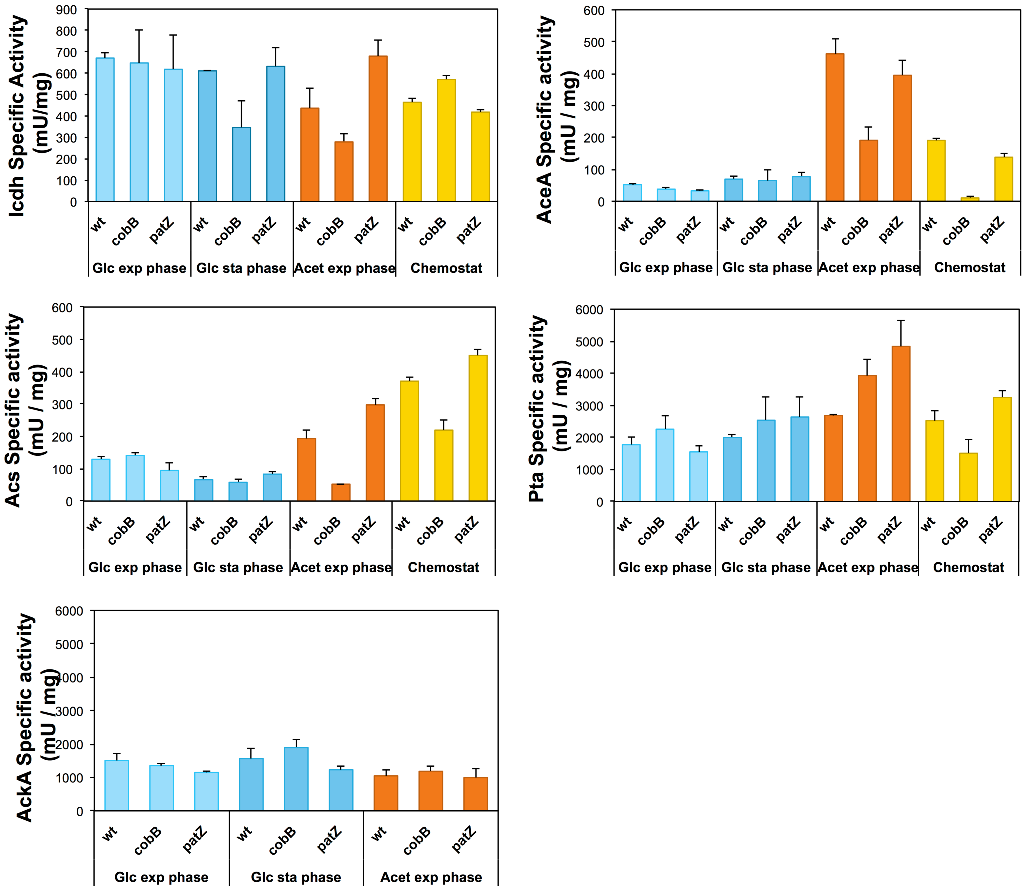

Supplementary Figure 6. Isocitrate dehydrogenase (Icdh), isocitrate lyase (AceA), acetyl-CoA synthetase (Acs), phosphotransacetylase (Pta) and acetate kinase (AckA) enzyme activities measured in cell crude extracts of *E. coli* BW25113 and its knockouts strains grown in glucose batch cultures, acetate batch cultures and glucose limited chemostat cultures.
