# Supplementary material for: Protein acetylation affects acetate metabolism, motility and acid stress response in Escherichia coli
Source: Mol Syst Biol. 2014 Nov 28;10(11):762. doi: 10.15252/msb.20145227 (PMC4299603; doi:10.15252/msb.20145227)
Supplement: Supplementary file 7 — Supplementary Figure S7 [file msb0010-0762-sd7.pdf]

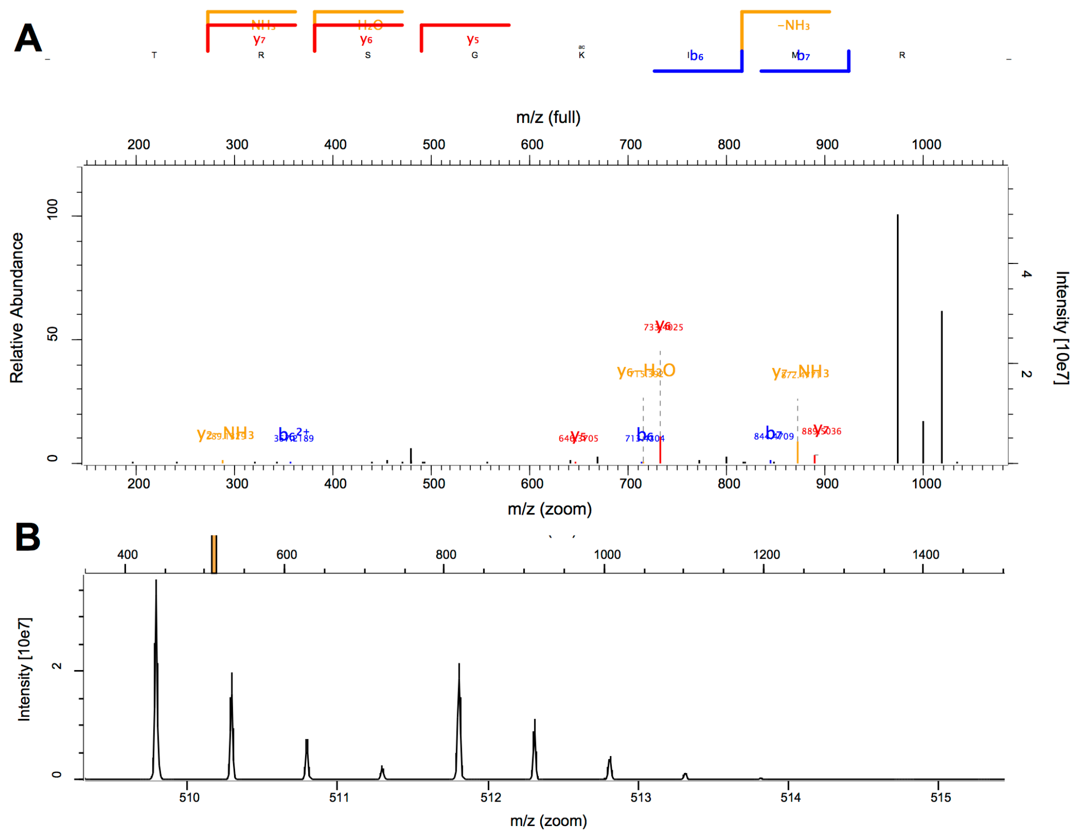

Supplementary Figure 7. The peptide containing the acetylated lysine 609 found in a different Maxquant analysis, where lysine dimethylation was set as variable modification. **(A)** Fragmented peptide containing lysine 609 in chemostat cultures. **(B)** MS1 scan showing two of the three labeling states of the peptide shown in A. Only the light (wild type) and the medium (*cobB* mutant) states are present in all the MS1 scan checked, which is consistent with lack of acetylation in the *patZ* mutant.
