# Supplementary material for: Protein acetylation affects acetate metabolism, motility and acid stress response in Escherichia coli
Source: Mol Syst Biol. 2014 Nov 28;10(11):762. doi: 10.15252/msb.20145227 (PMC4299603; doi:10.15252/msb.20145227)
Supplement: Supplementary file 8 — Supplementary Figure S8 [file msb0010-0762-sd8.pdf]

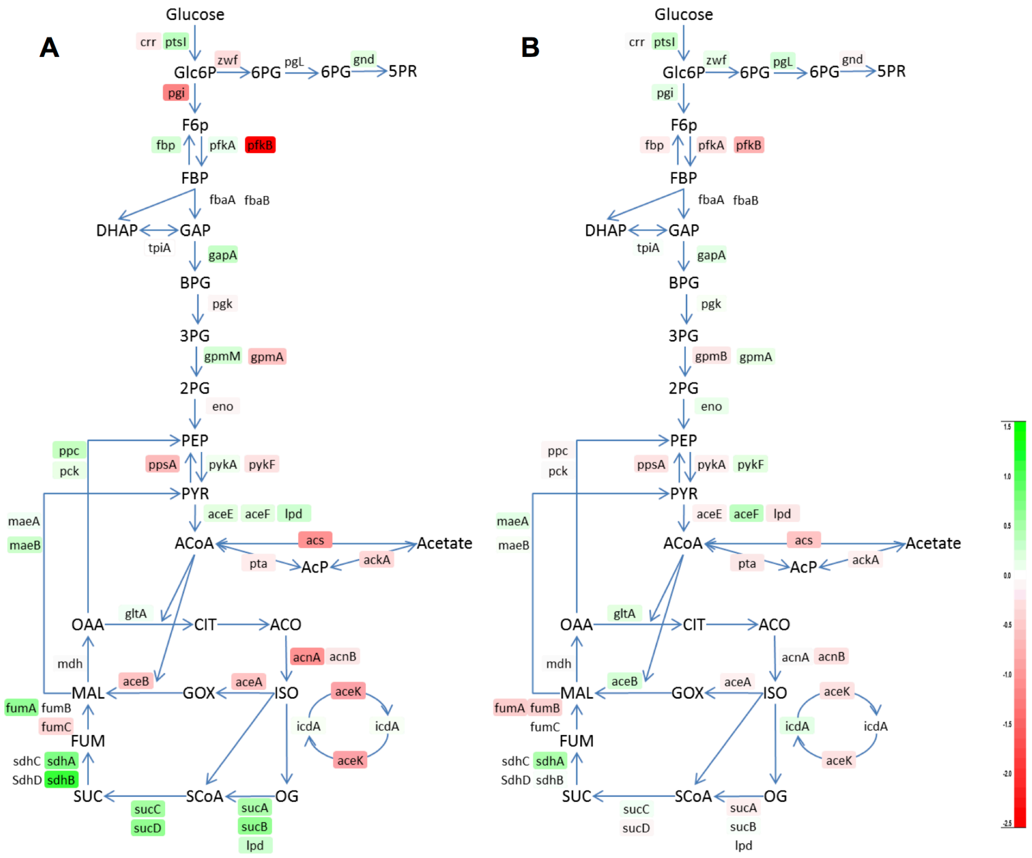

Supplementary Figure 8. Central metabolism proteins relative abundance ($\log_2$ ratio) of the **(A)** Δ*cobB* and **(B)** Δ*patZ* mutants in chemostat cultures. Protein concentrations are normalized against concentrations in the wild type (wt) strain grown under the same conditions. Relative abundance is represented by the color scale shown on the right side of the figure.
