# Supplementary material for: Protein acetylation affects acetate metabolism, motility and acid stress response in Escherichia coli
Source: Mol Syst Biol. 2014 Nov 28;10(11):762. doi: 10.15252/msb.20145227 (PMC4299603; doi:10.15252/msb.20145227)
Supplement: Supplementary file 9 — Supplementary Figure S9 [file msb0010-0762-sd9.pdf]

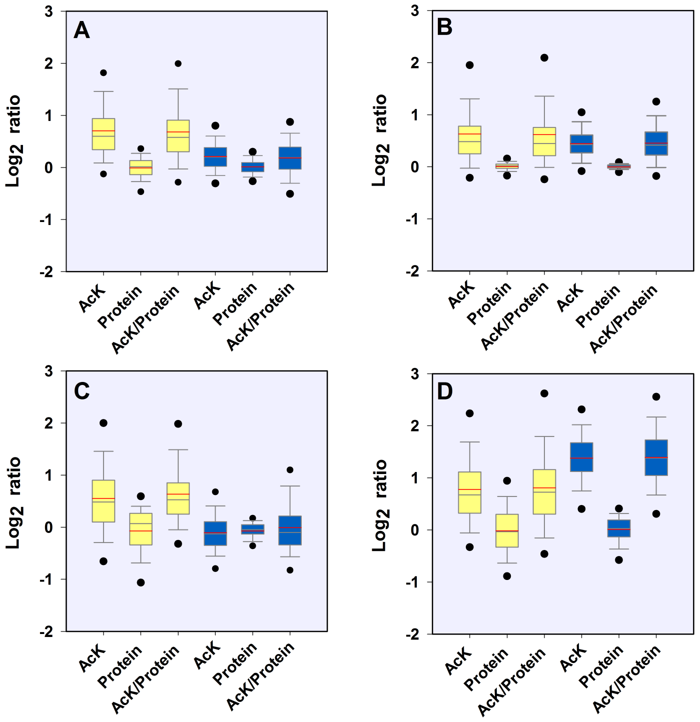

Supplementary Figure 9. Box plot of the distribution of the ratios of peptide acetylation (AcK), protein abundance (protein) and the ratios acetylated peptides normalized by protein abundance (AcK/protein) of the *cobB* mutant/wild type (yellow) and the *patZ* mutant/wild type (blue). Experimental conditions were: (A) glucose batch cultures exponential and (B) stationary phase, (C) low dilution rate glucose limited chemostat cultures and (D) acetate batch cultures. Red and grey lines represent median and average respectively.
