# Supplementary material for: Protein acetylation affects acetate metabolism, motility and acid stress response in Escherichia coli
Source: Mol Syst Biol. 2014 Nov 28;10(11):762. doi: 10.15252/msb.20145227 (PMC4299603; doi:10.15252/msb.20145227)
Supplement: Supplementary file 10 — Supplementary Figure S10 [file msb0010-0762-sd10.pdf]

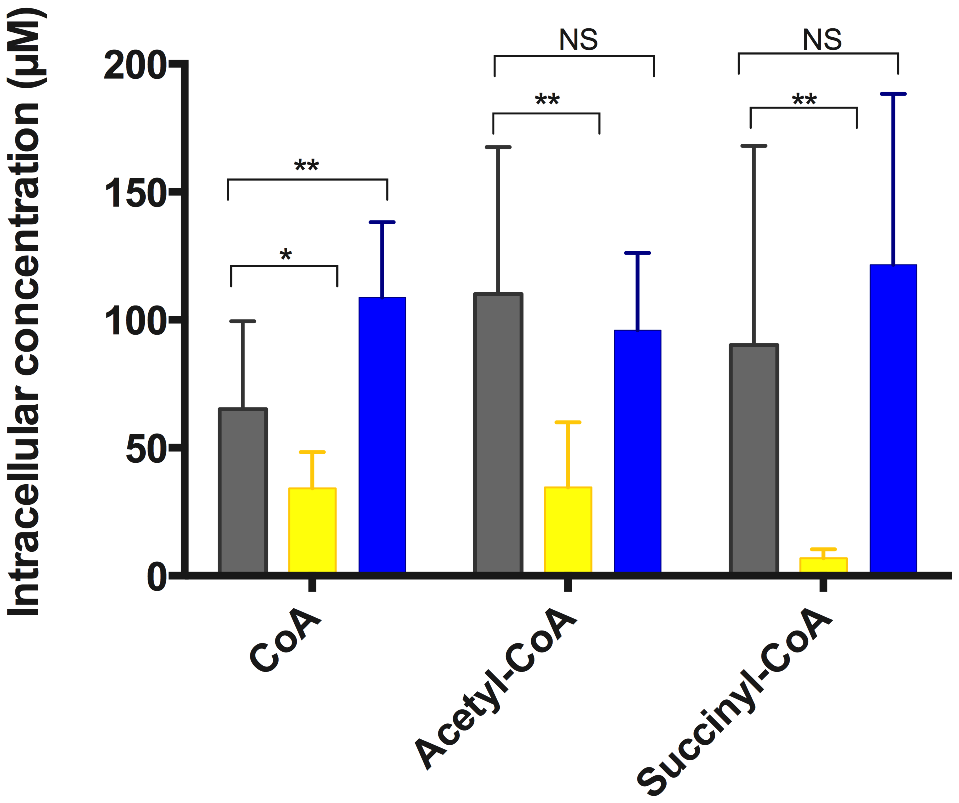

Supplementary Figure 10. Intracellular concentration of coenzyme A, acetyl CoA, and succinyl-CoA in *E. coli* BW25113 (grey bars) and its knockout derivatives, Δ*cobB* (yellow bars) and Δ*patZ* mutant (blue bars). Cultures were grown in acetate minimal medium and harvested during mid-exponential phase. Briefly, 8 ml culture broth of OD 1 was harvested by rapid filtration through 0.45 µM filter membranes, washed with 8 ml pre-warmed 10 mM ammonium carbonate buffer pH 7.2 and immediately extracted by transferring the cell pellet with the membranes into 4 ml acetonitrile:methanol:ddH2O (2:1:1, v/v) for 1 h and occasional shaking at -20°C. After removing precipitates by centrifugation at 16000xg and 4°C for 5 min, extracts were dried in a speed-vac for 4h, resuspended in 100 µl ddH2O and analyzed on a Thermo TSQ Quantum Ultra triple quadrupole instrument (Thermo Fisher Scientific, Waltham MA, United States) as described previously (Zimmermann et al, 2013). Dilution series of standards for quantification were spiked to fully ^{13}C-labelled *E. coli* wild type extracts and processed as described above. The mean and standard deviation of at least 5 replicates are represented in the plot. Statistics: 2-tailed t-test was performed * represents a p-value ≤0.01, ** p-value ≤0.05 and NS (non significant, p-value >0.1)
