# Supplementary material for: Protein acetylation affects acetate metabolism, motility and acid stress response in Escherichia coli
Source: Mol Syst Biol. 2014 Nov 28;10(11):762. doi: 10.15252/msb.20145227 (PMC4299603; doi:10.15252/msb.20145227)
Supplement: Supplementary file 11 — Supplementary Table S1 [file msb0010-0762-sd11.pdf]

Suppl. Table 1. Number of lysine acetylated peptides and proteins in all replicates and conditions assayed.

acK peptides	Replicate1	Replicate2	Replicate3	Replicate4	All
Glucose batch exponential phase	294	725	1124	501	**1486**
Glucose batch stationary phase	840	939	778	958	**1674**
Acetate batch cultures	658	790	1090	1233	**1867**
Glucose chemostat cultures	243	104	619	731	**1001**
**All conditions**					**2502**

acK proteins	Replicate1	Replicate2	Replicate3	Replicate4	All
Glucose batch exponential phase	177	370	366	179	**444**
Glucose batch stationary phase	392	451	288	336	**508**
Acetate batch cultures	331	401	366	408	**565**
Glucose chemostat cultures	147	75	229	270	**330**
**All conditions**					**809**
