# Supplementary material for: Protein acetylation affects acetate metabolism, motility and acid stress response in Escherichia coli
Source: Mol Syst Biol. 2014 Nov 28;10(11):762. doi: 10.15252/msb.20145227 (PMC4299603; doi:10.15252/msb.20145227)
Supplement: Supplementary file 12 — Supplementary Table S2 [file msb0010-0762-sd12.pdf]

**Supplem Table 2.** Proteins which activity in acetate cultures might be compromised in the *cobB* mutant.

| Protein | Protein residue and experimental evidence | References |
|---|---|---|
| Fructose-1,6-bisphosphatase class 1 (Fbp) | • K269: highly conserved. Belongs to catalytic site.<br>• Site specific mutation K269A reduces 20-fold the affinity of the enzyme for its substrate and decreases 500-fold affinity for the competitive inhibitor fructose 2,6-bisphosphate<br>• Higher acetylation of this residue in the Δ*cobB* mutant might affect gluconeogenesis. | (el-Maghrabi *et al*, 1992) |
| RNA polymerase sigma subunit (RpoD) | • K557: belongs to an helix-turn helix domain responsible for the interaction with the -35 box of gene promoters.<br>• It is not clear if this residue interacts directly with the DNA backbone, but the abundance of positively charged amino acids in this domain, suggests that it may play a role in the fine tuning of transcription initiation in *E. coli*. | (Paget & Helmann, 2003) |
| Ribosome-associated inhibitor A protein (Rai) | • K87: belongs a protein region that blocks the P-site (peptidyl-tRNA site) of the ribosome.<br>• Regulates translation in stationary phase in *E. coli*. It can block the dimer formation of 70S, decreasing the translation rate. This protein has two different regions that block the A-site (aminoacyl-tRNA site) and P-site (peptidyl-tRNA site) of the ribosome blocking translation. Both regions are particularly enriched with positively charged amino acids. | (Agafonov *et al*, 1999; Maki *et al*, 2000). |
| Long chain fatty acid CoA ligase (FadD) | • K543.<br>• When it is acetylated in its active site, as occurs on Acs, acyl-CoA synthase activity is abolished.<br>• The acetylation ratio was 35-fold higher in the *cobB* mutant than in the *patZ* mutant (not in all replicates).<br>• Regulation of FadD by lysine acetylation has also been described in other organisms like *Rhodopseudomonas palustris*. | (Crosby *et al*, 2010) |
| Glyceraldehyde-3-phosphate dehydrogenase (GapA) | • K184: necessary for $NAD^+$ binding and activity.<br>• It was found to be more acetylated only in the *cobB* mutant.<br>• The activity of this protein is essential under glycolitic and gluconeogenic conditions. | (Kuhn *et al*, 2014)<br>(Seta *et al*, 1997) |
