# Supplementary material for: Protein acetylation affects acetate metabolism, motility and acid stress response in Escherichia coli
Source: Mol Syst Biol. 2014 Nov 28;10(11):762. doi: 10.15252/msb.20145227 (PMC4299603; doi:10.15252/msb.20145227)
Supplement: Supplementary file 13 — Supplementary Table S3 [file msb0010-0762-sd13.pdf]

Suppl. Table 3. Lysine acetylated proteins related to transcription in *E. coli* (GO:0006351 and GO:0006355). Transcription factors marked with an asterisk are transcriptional regulators of flagella biosynthesis and chemotaxis and the ones marked with a cross are transcriptional regulators of acid stress response. Red symbols are four transcriptional repressors and green symbols for transcriptional activators**.**

Gene name	Uniprot ID	Number acetylation sites	Acetylated lysine position in the protein
agaR	P0ACK2	1	93
appY	P05052	2	47,55
arcA	P0A9Q1	3	187,195,160
arcB	P0AEC3	1	93
argR	P0A6D0	3	15,19,6
cheY	P0AE67	1	126
cpxR	P0AE88	1	219
crl	P24251	2	15,88
crp*†	P0ACJ8	3	101,153,167
csgD	P52106	1	201
cspC	P0A9Y6	3	9,15,59
cspE	P0A972	1	9
cysB	P0A9F3	1	320
dosP	P76129	1	95
ebgR	P06846	1	319
evgA	P0ACZ4	1	159
fis	P0A6R3	2	25,32
frlR	P45544	1	104
fur*	P0A9A9	1	117
glnB	P0A9Z1	2	85,90
greA	P0A6W5	2	63,116
gyrA	P0AES4	10	239,253,276,282,308,465,473516,573,671
gyrB	P0AES6	4	231,299,339,592
Hns*†	P0ACF8	5	6,57,120,128,136
hupA	P0ACF0	6	3,13,18,67,83,86
hupB	P0ACF4	5	3,9,18,67,86
ihfA*	P0A6X7	3	20,86,97
ihfB*	P0A6Y1	3	3,69,81
kdgR	P76268	1	170
lrp	P0ACJ0	4	10,25,36,129
malY	P23256	1	261
mprA	P0ACR9	1	97
nadR	P27278	1	47
nagC	P0AF20	1	63
narL	P0AF28	1	77
nusA	P0AFF6	2	117,243
nusB	P0A780	3	67,94,121
nusG	P0AFG0	2	106,121
ompR*	P0AA16	1	184
pcnB	P0ABF1	1	396
pepA	P68767	3	6,90,216
rcsB*†	P69407	2	125,154
relE	P0C077	1	13
rho	P0AG30	4	100,105,123,367
rplD	P60723	5	106,123,132,137,166
rpoA	P0A7Z4	5	95,246,291,297,304
rpoB	P0A8V2	19	115,191,236,279,639,844,890900,909,914,954,988,991,1027,1065,1133,1140,1200,1242
rpoC	P0A8T7	15	13,39,50,66,74,87,570603,649,850,959,983,996,1072,1132
rpoE	P0AGB6	1	16
rpoZ	P0A800	1	35
rpsD	P0A7V8	7	8,31,33,77,156,177,183
rsd	P0AFX4	2	17,79
rtcR	P38035	1	206
seqA	P0AFY8	1	60
stpA	P0ACG1	2	66,98
wrbA	P0A8G6	2	37,50
ydjF	P77721	2	80,158
yebC	P0A8A0	1	215
yeeN	P0A8A2	2	66,116
ygbI	P52598	1	188
yhaJ	P67660	1	62
yhaV	P64594	1	87
yiaU	P37682	1	7
