# Supplementary material for: Protein acetylation affects acetate metabolism, motility and acid stress response in Escherichia coli
Source: Mol Syst Biol. 2014 Nov 28;10(11):762. doi: 10.15252/msb.20145227 (PMC4299603; doi:10.15252/msb.20145227)
Supplement: Supplementary file 14 — Supplementary Table S4 [file msb0010-0762-sd14.pdf]

Suppl. Table 4. Metabolic fluxes of *Escherichia coli* and its knockout mutants (ΔcobB, ΔpatZ, ΔcobBΔaceK and ΔpatZΔaceK) grown in glucose batch and glucose limited chemostat cultures. All metabolic fluxes are normalized to 100% of glucose uptake rate (absolute values are expressed in: mmol g^{-1} h^{-1}.

	**Glucose batch cultures**					**Glucose chemostat cultures (D=0.2h^{-1})**				
	**wt**	**Δ*cobB***	**Δ*patZ***	**Δ*cobB*Δ*aceK***	**Δ*patZ*Δ*aceK***	**wt**	**Δ*cobB***	**Δ*patZ***	**Δ*cobB*Δ*aceK***	**Δ*patZ*Δ*aceK***
glucose uptake	100 (8.66±0.21)	100 (9.08±0.06)	100 (8.17±0.01)	100 (8.4±0.26)	100 (7.95±0.02)	100 (2.73±0.03)	100 (3.55±0.09)	100 (2.7±0.07)	100 (2.55±0.04)	100 (2.86±0.08)
zwf	23.73±0.79	23.03±0.05	22.49±1.78	*24.59±1.45*	*22.68±1*	33.74±1.9	26.5±0.86	34.61±2.54	25.16±1.16	28.58±0.61
gnd	23.1±0.37	23.03±0.05	22.39±1.67	21.43±2.57	22.24±0.27	26.91±0.81	21.1±0.35	28.35±1.76	18.1±0.33	25.77±1.78
pgi	75.8±0.79	76.56±0.06	77.05±1.78	74.99±1.44	76.86±1	65.82±1.88	73.2±0.87	64.94±2.51	74.44±1.16	70.98±0.6
edd	0.63±0.75	0±0	0.1±0.17	3.16±3.73	0.44±0.77	6.83±1.63	5.41±0.91	6.26±4.28	7.06±1.45	2.8±2.29
pfk-fba-tpi	86.19±0.78	87.54±0.13	87.04±0.66	84.85±2.81	86.85±0.83	79.35±1.44	84.21±0.93	79.34±3.43	82.44±1.33	83.62±1.53
tktA	7.27±0.11	7.33±0.02	7.07±0.56	6.82±0.85	7.04±0.09	8.89±0.27	6.98±0.12	9.37±0.59	5.97±0.11	8.5±0.6
tktB	3.75±0.03	4.19±0.08	3.54±0.57	3.6±0.82	3.56±0.1	5.23±0.35	4.43±0.09	5.63±0.39	2.56±0.09	4.74±0.43
tal	7.27±0.11	7.33±0.02	7.07±0.56	6.82±0.85	7.04±0.09	8.89±0.27	6.98±0.12	9.37±0.59	5.97±0.11	8.5±0.6
gap-pgk	175.77±0.91	178.39±0.36	176.73±0.64	175.59±2.62	176.74±0.8	169.88±0.96	177.67±1.03	169.69±2.92	173.71±1.27	173.9±1.23
gpm-eno	162.75±1.13	166.85±0.66	163.77±0.61	163.92±2.4	164.01±0.77	157.7±0.47	168.86±1.15	157.29±2.4	162.25±1.08	161.12±1.08
pyk	128.61±1.87	135.65±2.32	129.54±0.5	132.97±2.12	130.28±0.68	182.98±6.53	180.78±5.01	182.97±3.1	145.42±8.09	141.34±6.24
ace-lpd	100.78±2.42	110.67±2.14	101.05±0.27	110.31±3.38	102.59±0.16	165.97±5.89	163.94±5.91	164.77±2.08	127.97±5.74	114.41±4.01
glt-acn	29.17±4.2	26.6±4.16	24.13±2.97	19.79±6.49	26.03±0.36	89.39±2.28	93.04±3.45	87.36±2.24	69.1±5.31	78.08±2.8
icd	29.17±4.2	26.6±4.16	24.13±2.97	19.79±6.49	26.03±0.36	30.97±3.45	54.57±1.86	28.4±2.18	52.46±8	61.79±7.73
suc-sdh	16.68±4.52	15.35±4.46	11.58±3.02	8.45±6.91	13.67±0.39	20.13±3.47	44.37±1.27	17.42±1.95	40.91±8.72	48.51±7.63
fum	16.68±4.52	15.35±4.46	11.58±3.02	8.45±6.91	13.67±0.39	78.54±2.13	82.84±3.77	76.38±1.95	57.55±6.24	64.79±2.65
mdh	16.68±4.52	14.75±5.26	11.58±3.02	8.45±6.91	13.67±0.39	134.81±8.25	121.31±8.62	133.49±2.24	72.23±10.44	80.83±4.06
mae	0±0	0.61±1.05	0±0	0±0	0±0	2.14±2.26	0±0	1.85±1.55	1.96±3.39	0.25±0.32
pck	6.75±1.16	7.64±3.25	8.65±1.7	9.56±2.85	6.07±1.32	41.06±4.72	43.6±2.48	39.78±1.72	24.14±5.69	30.84±1.27
pcc	33.4±0.52	32.15±1.74	35.38±1.64	33.68±2.1	32.38±1.28	8.17±2.07	26.2±3.19	6.37±0.48	33.8±2.66	42.68±6.16
acetate secretion	51.08±1.26	65.85±1.55	56.47±3.17	72.14±2.49	56.5±0.15	0±0	18.11±3.73	0±0	24.46±1.18	0±0
glyoxylate shunt	0±0	0±0	0±0	0±0	0±0	58.41±4.56	38.47±4.87	58.96±0.59	16.63±4.96	16.29±5.44
transhydrogenase	64.43±6.97	51.87±7.27	70.99±6.69	61.35±10.94	67.2±1.95	50.43±9.15	0±0	53.28±11.16	34.56±8.76	27.2±13.87
respiration	140.65±11.49	150.01±11.36	130.22±7.41	133.47±16.36	135.85±1.21	269.37±9.24	301.01±8.27	264.18±7.56	227.95±13.51	236.7±9.14
growth	8.67±0.22	7.61±0.19	8.58±0.02	7.74±0.25	8.43±0.02	8.16±0.47	5.49±0.17	8.31±0.48	7.45±0.01	8.24±0.47
