# Supplementary material for: Protein acetylation affects acetate metabolism, motility and acid stress response in Escherichia coli
Source: Mol Syst Biol. 2014 Nov 28;10(11):762. doi: 10.15252/msb.20145227 (PMC4299603; doi:10.15252/msb.20145227)
Supplement: Supplementary file 15 — Supplementary Table S5 [file msb0010-0762-sd15.pdf]

Suppl. Table 5. Up regulated genes (log_2) in the *cobB* mutant compared with the wild type in glucose exponential phase cultures (FDR<0.05).

Genes	Fold change	Description
**Policistronic operons**		
fliLMNOPQR		
fliL	3.275	flagellar basal body-associated protein FliL
fliM	3.090	flagellar motor switch protein FliM
fliN	3.150	flagellar motor switch protein FliN
fliO	2.798	flagellar biosynthesis protein FliO
fliP	2.597	flagellar biosynthesis protein FliP
fliQ	2.294	flagellar biosynthesis protein FliQ
fliR	2.264	flagellar biosynthesis protein FliR
fliFGIJK		
fliF	3.081	flagellar MS-ring protein
fliG	3.200	flagellar motor switch protein G
fliI	2.849	flagellum-specific ATP synthase
fliJ	3.080	flagellar biosynthesis chaperone
fliK	2.797	flagellar hook-length control protein
FlgBCDEFGHIJKL		
flgB	3.617	flagellar basal body rod protein FlgB
flgC	3.484	flagellar basal body rod protein FlgC
flgD	3.354	flagellar basal body rod modification protein
flgE	3.477	flagellar hook protein
flgF	3.477	flagellar component of cell-proximal portion of basal-body rod
flgG	3.325	flagellar basal body rod protein FlgG
flgH	3.274	flagellar basal body L-ring protein
flgI	3.665	flagellar basal body P-ring protein
flgJ	3.293	flagellar rod assembly protein
flgK	3.853	flagellar hook-associated protein FlgK
flgL	3.526	flagellar hook-associated protein FlgL
FlhBAE		
flhB	2.341	flagellar biosynthesis protein FlhB
flhA	2.058	flagellar biosynthesis protein FlhA
flhE	2.322	flagellar protein
FlgAMN		
flgA	3.158	flagellar basal body P-ring biosynthesis protein FlgA
flgM	3.115	anti-sigma28 factor FlgM
flgN	3.447	flagella synthesis protein FlgN
FliAZ		
fliA	3.693	flagellar biosynthesis sigma factor
fliZ	3.572	flagella biosynthesis protein FliZ
tar-tap-cheRYZ		
tar	4.561	methyl-accepting chemotaxis protein II
tap	4.123	methyl-accepting protein IV
cheR	3.533	chemotaxis methyltransferase CheR
cheB	3.583	chemotaxis-specific methylesterase
cheY	3.618	chemotaxis regulatory protein CheY
cheZ	3.505	chemotaxis regulator CheZ
fliDST		
fliD	4.380	flagellar capping protein
fliS	3.981	flagellar protein FliS
fliT	3.054	flagellar biosynthesis protein FliT
motAB-CheAW		
motA	3.916	flagellar motor protein MotA
motB	4.004	flagellar motor protein MotB
cheA	4.175	chemotaxis protein CheA
cheW	4.149	purine-binding chemotaxis protein
flhCD		
flhC	1.925	transcriptional activator FlhC
flhD	1.921	transcriptional activator FlhD
**Monocistronic operons**		
fliC	4.808	flagellar filament structural protein (flagellin)
fliE	2.907	flagellar hook-basal body protein FliE
tsr	3.883	methyl-accepting chemotaxis protein I
ycgR	3.717	hypothetical protein
yhjH	3.613	EAL domain-containing protein
flxA	3.519	Qin prophage; predicted protein
yecR	2.813	hypothetical protein
ymdA	2.688	hypothetical protein
yjcZ	2.630	hypothetical protein
ynjH	2.477	hypothetical protein
aer	1.467	aerotaxis receptor
