# Supplementary material for: Protein acetylation affects acetate metabolism, motility and acid stress response in Escherichia coli
Source: Mol Syst Biol. 2014 Nov 28;10(11):762. doi: 10.15252/msb.20145227 (PMC4299603; doi:10.15252/msb.20145227)
Supplement: Supplementary file 16 — Supplementary Table S6 [file msb0010-0762-sd16.pdf]

Suppl. Table 6. Down regulated genes (log_2) in the *cobB* mutant compared with the wild type in glucose exponential phase cultures (FDR<0.05).

Genes	fold change	Description
**policistronic operons**		
gadBC		
gadB	-1.870	glutamate decarboxylase isozyme
gadC	-1.259	acid sensitivity protein
hdeAB-yhiD		
hdeA	-1.819	acid-resistance protein
hdeB	-1.627	acid-resistance protein
entCEBAH		
entC	-1.531	isochorismate synthase
entE	-1.804	2,3-dihydroxybenzoate-AMP ligase component of enterobactin synthase multienzyme complex
entB	-1.191	2,3-dihydro-2,3-dihydroxybenzoate synthetase
gadAXW		
gadA	-1.156	glutamate decarboxylase isozyme
gadX	-1.718	DNA-binding transcriptional regulator GadX
ybaST		
ybaS	-1.548	glutaminase
puuRDCBE		
puuR	-1.263	DNA-binding transcriptional repressor PuuR
puuD	-1.288	gamma-glutamyl-gamma-aminobutyrate hydrolase
puuC	-1.465	gamma-glutamyl-gamma-aminobutyraldehyde dehydrogenase
puuB	-1.420	gamma-Glu-putrescine oxidase, FAD
talA-tktB		
tktB	-1.268	transketolase
yeaGH		
yeaG	-1.180	hypothetical protein
acs-yjcH-actP		
acs	-1.153	acetyl-CoA synthetase
gadE-mdtEF		
gadE	-1.149	hypothetical protein
fadBA		
fadB	-1.126	multifunctional fatty acid oxidation complex subunit alpha
**monocistronic operons**		
mscS	-1.160	mechanosensitive channel MscS
katE	-1.114	hydroperoxidase II
yciE	-1.114	hypothetical protein
entF	-1.112	enterobactin synthase multienzyme complex component, ATP-dependent
hchA	-1.103	chaperone protein HchA
pfkB	-1.760	6-phosphofructokinase 2
fiu	-1.477	catecholate siderophore receptor Fiu
hdeD	-1.376	acid-resistance membrane protein
cirA	-1.241	colicin I receptor
ydiZ	-1.153	hypothetical protein
ydhS	-1.131	hypothetical protein
ygiW	-1.487	hypothetical protein
