# Supplementary material for: Protein acetylation affects acetate metabolism, motility and acid stress response in Escherichia coli
Source: Mol Syst Biol. 2014 Nov 28;10(11):762. doi: 10.15252/msb.20145227 (PMC4299603; doi:10.15252/msb.20145227)
Supplement: Supplementary file 17 — Supplementary Table S7 [file msb0010-0762-sd17.pdf]

Suppl. Table 7. Up regulated genes (log_2) in the *cobB* mutant compared with the wild type in glucose chemostat cultures (FDR<0.05).

Genes	Fold change	Description
**policistronic operons**		
tar-tap-cheRBYZ		
tar	4.448	methyl-accepting chemotaxis protein II
tap	4.204	methyl-accepting protein IV
cheR	3.524	chemotaxis methyltransferase CheR
cheB	3.344	chemotaxis-specific methylesterase
cheY	4.200	chemotaxis regulatory protein CheY
cheZ	3.538	chemotaxis regulator CheZ
fliDST		
fliD	4.944	flagellar capping protein
fliS	4.306	flagellar protein FliS
fliT	2.994	flagellar biosynthesis protein FliT
motAB-cheAW		
motA	3.933	flagellar motor protein MotA
motB	3.500	flagellar motor protein MotB
cheA	3.834	chemotaxis protein CheA
cheW	4.397	purine-binding chemotaxis protein
flgKL		
flgK	4.355	flagellar hook-associated protein FlgK
flgL	3.764	flagellar hook-associated protein FlgL
fliAZY		
fliA	4.497	flagellar biosynthesis sigma factor
fliZ	4.380	flagella biosynthesis protein FliZ
fliY		
flgBCDEFGHIJ		
flgB	3.415	flagellar basal body rod protein FlgB
flgC	3.185	flagellar basal body rod protein FlgC
flgD	3.626	flagellar basal body rod modification protein
flgE	2.778	flagellar hook protein
flgF	3.880	flagellar component of cell-proximal portion of basal-body rod
flgG	3.394	flagellar basal body rod protein FlgG
flgH	3.134	flagellar basal body L-ring protein
flgI	3.279	flagellar basal body P-ring protein
flgJ	2.981	flagellar rod assembly protein
flgAMN		
flgA	3.357	flagellar basal body P-ring biosynthesis protein FlgA
flgM	4.290	anti-sigma28 factor FlgM
flgN	3.864	flagella synthesis protein FlgN
fliLMNOPQ		
fliL	3.113	flagellar basal body-associated protein FliL
fliM	2.961	flagellar motor switch protein FliM
fliN	3.555	flagellar motor switch protein FliN
fliO	2.285	flagellar biosynthesis protein FliO
fliP	1.437	flagellar biosynthesis protein FliP
fliQ	1.676	flagellar biosynthesis protein FliQ
fliFGHIJK		
fliF	2.918	flagellar MS-ring protein
fliG	3.159	flagellar motor switch protein G
fliH		
fliI	2.785	flagellum-specific ATP synthase
fliJ	2.764	flagellar biosynthesis chaperone
fliK	2.704	flagellar hook-length control protein
yjfAZ		
yjcZ	2.754	hypothetical protein
flhBA		
flhB	2.248	flagellar biosynthesis protein FlhB
flhA	2.072	flagellar biosynthesis protein FlhA
flhCD		
flhC	2.873	transcriptional activator FlhC
flhD	2.844	transcriptional activator FlhD
fimAICDFGH		
fimA	2.177	major type 1 subunit fimbrin (pilin)
codAB		
codA	1.524	cytosine deaminase
codB		
modABC		
modC	1.466	molybdate transporter ATP-binding protein
upp-uraA		
upp	1.454	uracil phosphoribosyltransferase
uraA		

purMN		
purM	1.414	phosphoribosylaminoimidazole synthetase
ykfBFXGHWI		
ykfB	2.088	CP4-6 prophage; predicted protein
nuoMN		
nuoM	1.560	NADH dehydrogenase subunit M
**monocistronic operons**		
fliC	4.418	flagellar filament structural protein (flagellin)
tsr	3.771	methyl-accepting chemotaxis protein I
fliE	1.591	flagellar hook-basal body protein FliE
ndk	1.621	nucleoside diphosphate kinase
aer	2.673	aerotaxis receptor
ycfQ	1.795	hypothetical protein
ycgR	3.577	hypothetical protein
yecR	2.139	hypothetical protein
ymdA	2.819	hypothetical protein
ynjH	2.970	hypothetical protein
flxA	3.543	Qin prophage; predicted protein
yhjH	3.395	EAL domain-containing protein
