# Supplementary material for: Protein acetylation affects acetate metabolism, motility and acid stress response in Escherichia coli
Source: Mol Syst Biol. 2014 Nov 28;10(11):762. doi: 10.15252/msb.20145227 (PMC4299603; doi:10.15252/msb.20145227)
Supplement: Supplementary file 18 — Supplementary Table S8 [file msb0010-0762-sd18.pdf]

Suppl. Table 8. Down regulated genes (log_2) in the *cobB* mutant compared with the wild type in glucose chemostat cultures (FDR<0.05).

Genes	Fold change	Description
**policistronic operons**		
prpBCDE		
prpB	-2.866	2-methylisocitrate lyase
prpC	-2.869	methylcitrate synthase
prpD	-3.099	2-methylcitrate dehydratase
prpE	-2.729	propionate--CoA ligase
lsrACDHFG-tam		
lsrA	-2.340	putative ATP-binding component of a transport system
hdeAB-yhiD		
hdeA	-2.139	acid-resistance protein
hdeB	-2.298	acid-resistance protein
ydcSTUV		
ydcS	-1.640	putative transport protein
yddLKJ		
yddK	-1.540	predicted protein
gadBC		
gadB	-1.530	glutamate decarboxylase isozyme
gadE-mdtEF		
gadE	-1.465	hypothetical protein
yeaGH		
yeaG	-1.458	hypothetical protein
yaiXO		
yaiX	-1.368	insertion element IS2 transposase InsD
ostBA		
otsB	-1.346	trehalose-6-phosphate phosphatase
yjbLM		
yjbL	-1.240	predicted protein
lsrRK		
lsrR	-1.230	putative SORC-type transcriptional regulator
yjdKO		
yjdK	-1.220	hypothetical protein
yhcADE		
yhcE	-1.203	pseudogene
gadAXW		
gadW	-1.192	putative ARAC-type regulatory protein
yciGFE		
yciG	-1.151	hypothetical protein
elfDCG-ycbUVF		
elfD	-1.119	putative chaperone
**monocistronic operons**		
ryjA	-2.368	ncRNA
ryhB	-1.415	ncRNA
rprA	-1.351	ncRNA
micF	-1.311	ncRNA
rpsV	-1.113	30S ribosomal subunit S22
pfkB	-1.715	6-phosphofructokinase 2
aldB	-1.598	aldehyde dehydrogenase B (lactaldehyde dehydrogenase)
ecnB	-1.624	entericidin B membrane lipoprotein
csiE	-1.570	stationary phase inducible protein CsiE
katE	-1.349	hydroperoxidase II
ycgB	-1.534	SpoVR family protein
yafF	-1.461	H repeat-containing protein
insB-1	-1.301	IS1 transposase B
ompC	-1.289	outer membrane porin protein C
hdeD	-1.238	acid-resistance membrane protein
ldrA	-1.162	toxic polypeptide, small
ldrB	-1.121	toxic polypeptide, small
yneL	-1.111	hypothetical protein
yodD	-1.101	hypothetical protein
yjfN	-2.204	hypothetical protein
ygiW	-1.934	hypothetical protein
ydcH	-1.869	hypothetical protein
yebV	-1.371	hypothetical protein
yiaG	-1.281	putative transcriptional regulator
yqgD	-1.275	hypothetical protein
yjiC	-1.261	predicted protein
yohC	-1.252	hypothetical protein
phnB	-1.184	hypothetical protein
Z0115	-1.157	hypothetical protein
ydiZ	-1.150	hypothetical protein
ytfK	-1.128	hypothetical protein
