# Supplementary material for: Protein acetylation affects acetate metabolism, motility and acid stress response in Escherichia coli
Source: Mol Syst Biol. 2014 Nov 28;10(11):762. doi: 10.15252/msb.20145227 (PMC4299603; doi:10.15252/msb.20145227)
Supplement: Supplementary file 19 — Supplementary Table S9 [file msb0010-0762-sd19.pdf]

Suppl. Table 9. All strains and plasmids used in this study.

Strains	Relevant genotype	Source
E. coli BW25113	*lacI* ^{q} *rrnB*$_{T14}$ D*lacZ*$_{WJ16}$ *hsdR*514 D(*araBAD*)$_{AH33}$ D(*rhaBAD*)$_{LD78}$	Keio Collection(Baba *et al*, 2006)
**BW25113 derivatives**		
Δ*cobB*	[BW25113] *cobB*:*kan*	Keio Collection(Baba *et al*, 2006)
Δ*patZ*	[BW25113] *patZ*:*kan*	Keio Collection(Baba *et al*, 2006)
Δ*cobB*Δ*aceK*	[BW25113] *cobB*:*frt aceK:kan*	This study(Datsenko & Wanner, 2000)
Δ*patZ* Δ*aceK*	[BW25113] *patZ*:*frt aceK:kan*	This study(Datsenko & Wanner, 2000)
Δ*rcsB*	[BW25113] *rcsB*:*kan*	Keio Collection(Baba *et al*, 2006)
E. coli BL21 DE3 Δ*cobB*	F– ompT gal dcm lon hsdSB(rB- mB-) λ(DE3) cobB:kan	This study(Datsenko & Wanner, 2000)
E. coli DH10B	F- *mcr*A D(*mrr-hsd*RMS-*mcr*BC) phi;80*lac*ZDM15 D*lac*X74 *recA*1 *endA*1 *araD*139 D(*ara, leu*) 7697 *gal*U *gal*K l- *rps*L *nup*G<br>Host for cloning and plasmid propagation	Invitrogen
**Plasmids**		
pKD13	KanR/AmpR plasmid	Yale *E. coli* Genomic Resource Center
pKD46	AmpR plasmid that shows temperature-sensitive replication and thermal induction of FLP synthesis	Yale *E. coli* Genomic Resource Center
pCP20	AmpR/CmR plasmid that shows temperature-sensitive replication and thermal induction of FLP synthesis	Yale *E. coli* Genomic Resource Center
pBAD24	AmpR Arabinose promoter.	Invitrogen
pBAD24-*rcsB*	*rcsB* gene from *E. coli* BW25113	This study
pBAD24-*rcsB* K154R	*rcsB* substitution lysine 154 by arginine	This study
pBAD24-*rcsB* K154Q	*rcsB* substitution lysine 154 by glutamine	This study
pBAD24-*rcsB* K154E	*rcsB* substitution lysine 154 by glutamic	This study
pBAD24-*cobB*	*cobB* gene from *E. coli* BW25113	This study
pBAD24-*cobB*	*cobB* substitution histidine110 by tyrosine	This study
pCA24N-*cobB*H110Y	GFP (-)	Aska library(Kitagawa *et al*, 2005)
pCA24N-*acs*	GFP (-)	Aska library(Kitagawa *et al*, 2005)
pCA24N-*aceA*	GFP (-)	Aska library(Kitagawa *et al*, 2005)
