# Supplementary material for: Protein acetylation affects acetate metabolism, motility and acid stress response in Escherichia coli
Source: Mol Syst Biol. 2014 Nov 28;10(11):762. doi: 10.15252/msb.20145227 (PMC4299603; doi:10.15252/msb.20145227)
Supplement: Supplementary file 20 — Supplementary Table S10 [file msb0010-0762-sd20.pdf]

Suppl. Table 10. Primers used in this study.

		(5`→3`)
***cobB* KO**	fwd	GTGGTGCGGCCTTCCTACATCTAACCGATTAAACAACAGAGGTTGCTATGATTCCGGGGATCCGTCGACC
	rev	CCCCTTGCAGGCCTGATAAGCGTAGTGCATCAGGCAATGCTTCCCGCTTTTGTAGGCTGGAGCTGCTTCG
***aceK* KO**	fwd	CGTTTACGCCGCATCCGGCAATTCTCTGCTCCTGATGAGGGCGCTAAATGATTCCGGGGATCCGTCGACC
	rev	TGCGGAGAAAAATTATATGGAAGCTTTACTCAAAAAAGCATCTCCCCATATGTAGGCTGGAGCTGCTTCG
pBAD24-rcsB	fwd	GGTGGTTCTAGAAATGAACAATATGAACGTAATTATTG
	rev	GGTGGTAAGCTTTTAGTCTTTATCTGCCGGACTT
***rcsB* K154R**	fwd	CAAGCGTCTCTCGCCACGTGAGAGTGAAGTTCTG
	rev	CAGAACTTCACTCTCACGTGGCGAGAGACGCTTG
***rcsB* K154Q**	fwd	CAAGCGTCTCTCGCCACAGGAGAGTGAAGTTCTG
	rev	CAGAACTTCACTCTCCTGTGGCGAGAGACGCTTG
***rcsB* K154E**	fwd	GCGTCTCTCGCCAGAAGAGAGTGAAG
	rev	CTTCACTCTCTTCTGGCGAGAGACGC
pBAD24-cobB	fwd	GTTGTTTCTAGAAATGCTGTCGCGTCGGGGTC
	rev	GTTGTTAAGCTTTCAGGCAATGCTTCCCGCTTT
cobBH110Y	fwd	GAAATTCAGCCTAACGCCGCGTATCTTGCGCTGGCTAAACTG
	rev	CAGTTTAGCCAGCGCAAGATACGCGGCGTTAGGCTGAATTTC
