# Supplementary material for: Protein acetylation affects acetate metabolism, motility and acid stress response in Escherichia coli
Source: Mol Syst Biol. 2014 Nov 28;10(11):762. doi: 10.15252/msb.20145227 (PMC4299603; doi:10.15252/msb.20145227)
Supplement: Supplementary file 21 — Supplementary Information [file msb0010-0762-sd21.pdf]

Supplementary Material

Supplementary information

- *cobB* complementation in acetate minimal media with *pBAD-cobB* and *pBAD-cobB H110Y*.
- Extended description of regulation by lysine acetylation of the isocitrate node in *Escherichia coli.*
- Extended description of the effect of protein abundance on the detection of acetylation.
- Extended description of the analysis of Gene Ontologies of acetylated proteins.

Supplementary Methods

- Gene ontologies and quantile analysis
- Complementation experiment
- Western blotting.
- Deacetylation assays mass spectrometry.

Supplementary datasets (as Excel files)

Supplem data1 Protein lysine acetylation quantification data all conditions and replicates

Supplem data2 Significant acetylated lysines Δ*cobB and* Δ*patZ mutants* in acetate cultures

Supplem data3 Protein lysine acetylation quantification data all conditions and replicates normalized with the relative protein abundance.

Supplem data4 Relative protein abundance in glucose limited chemostat cultures (D=0.2 h^{-1})

Supplem data5 Relative gene expression (Microarray data) in glucose exponential phase and glucose limited chemostat cultures (D=0.2 h^{-1}).

Supplementary information.

***cobB* complementation in acetate minimal media with *pBAD-cobB* and *pBAD-cobB* H110Y.**

The knockout mutant Δ*cobB* was transformed with the plasmid containing the native *cobB* gene or the same gene encoding for an inactive version of the protein (For futher information see Suppl. methods). Both transformants and the wild type strain, also harbouring the empty vector, were grown overnight in LB media supplemented with acetate as carbon source and appropriate amount of antibiotics. Bacteria were cleaned three times with 0.9% NaCl and used for the inoculation of 25 mL of minimal medium with acetate as sole carbon source. Growth of all strains and mutants was followed during 25 hours. The Δ*cobB*+*pBAD-cobB* did not grow at all. Meanwhile, the mutant carrying the *cobB* inactive protein show a similar phenotype as the *cobB* mutant. (**Suppl. Fig. 4**). This shows that the phenotypic and proteomic effect observed in this mutant is caused by the absence of the deacetylase activity.

Extended description of regulation by lysine acetylation of the isocitrate node in *Escherichia coli.*

The reduced growth rate and biomass yield under acetate and chemostat conditions of the Δ*cobB* mutant indicate that acetate assimilation pathways could be affected. The glyoxylate shunt is essential for growth on acetate as the sole carbon source. In fact, the isocitrate node is an important regulation point of anabolism and catabolism. Isocitrate is a substrate for two enzymes, isocitrate dehydrogenase (Icd) and isocitrate lyase (AceA). Isocitrate dehydrogenase is part of the TCA cycle and its function is mainly catabolic, while isocitrate lyase is part of the glyoxylate shunt, a primarily anabolic pathway that shortcuts the TCA cycle.

The complex regulation of the isocitrate node in bacteria complicates the straightforward identification of the molecular target determining this metabolic shift. The different affinity of isocitrate dehydrogenase and isocitrate lyase towards its common substrate is the driving force explaining flux distributions at this node. Isocitrate dehydrogenase outcompetes for substrate due to its higher affinity, and a net flux through the glyoxylate shunt is only observed upon isocitrate accumulation. The reversible phosphorylation of isocitrate dehydrogenase by the action of AceK (isocitrate dehydrogenase phosphatase/kinase decreases flux through the TCA cycle (Borthwick *et al*, 1984; LaPorte & Koshland, 1983; LaPorte *et al*, 1984). It has been described that in *S. enterica* this metabolic node is also controlled by the acetylation of AceK (Wang *et al*, 2010).

In order to explore the functional consequences of metabolic enzymes acetylation contributing to acetate overflow and biomass yield, metabolic fluxes were determined in the *E. coli* mutants Δ*cobB and ΔpatZ,* on glucose batch cultures and low dilution rate glucose limited chemostat cultures **(Suppl. Table 1)**. Furthermore, in our proteomic study, acetylation of AceK was not detected. In order to demonstrate that the acetylation of AceK was not the causing the metabolic shift at the isocitrate node, metabolic fluxes the double (Δ*cobB*Δ*aceK*, Δ*patZ*Δ*aceK*) mutants were determined (**Suppl. Table 1**). In an *E. coli aceK* mutant, isocitrate dehydrogenase is permanently

active, , thus decreasing the flux through the glyoxylate shunt (Renilla *et al*, 2012). The deletion of *cobB* decreased flux in the bypass by half compared to the parent strain, while deletion of *patZ* exerted almost no effect on fluxes. Interestingly, the Δ*cobB*Δ*aceK* and Δ*patZ*Δ*aceK* mutants were more affected than the simple knockout strain Δ*cobB* **(Suppl. Table 1)**. Although the shunt was less active in both double mutants, the overflow of acetate and lower biomass yield were only observed in the Δ*cobB*Δ*aceK* mutant, as in the *cobB* mutant. Altogether, this shows that phosphorylation of isocitrate dehydrogenase by AceK is the main process regulating flux partitioning at the isocitrate node and that AceK is not affected by acetylation.

Extended description of the effect of protein abundance on the detection of acetylation.

The actual concentration (abundance) of proteins in the cell affects the probability of detecting their acetylated peptides. Therefore, acetylated peptides dataset might be biased towards abundant proteins. In order to test this, for each condition and biological replicate all proteins detected in the proteomic studies were grouped into five groups or quantiles depending on their intensities (where quantile Q1 groups the less abundant proteins and quantile Q5 the most abundant ones). Protein relative quantification was based on LC-MS runs with tryptic digests of whole protein extracts (*i.e.*, prior to immunoprecipitation of acetylated peptides). Subsequently, acetylated proteins were classified into the different proteome quantiles. The percentage of acetylated proteins in each quantile is represented in **Figure 3B**. It is evident that most of the acetylated proteins that have been identified in our study belong to proteins that are highly abundant in the proteome, *i.e.* quantiles Q4 and Q5. Therefore, the detection of proteins acetylated is biased by the abundance of the proteins in the whole proteome.

Extended description of Gene Ontologies analysis of acetylated proteins.

In order to test if Gene Ontology (GO) analysis was biased by protein abundance, we analyzed the different biological functions represented by proteins belonging to the previously described quantiles Q1-Q5. The abundance of the different GO terms was similar in each quantile, meaning that functions defined by GO terms are evenly distributed across the whole dynamic range of protein abundances. Moreover, these frequencies of GO terms were very similar to the frequencies at which they are represented in the whole proteome (**Supplementary Figure 5**).

Therefore, we can conclude that i) detection of protein acetylation is a function of protein abundance in the cell and ii) the frequency of GO terms in the set of acetylated proteins reflects their own frequency in the whole genome, and no specific function is over-represented (**Supplementary Figure 5**).

Supplementary Methods.

Gene ontologies data analysis and its relation with protein abundance

For each experimental replicate and condition, total protein extracts (prior to immunoprecipitation) were quantified by LC-MS/MS single runs. The proteins quantified were divided into 5 quantiles (Q1 to Q5) according to the intensity of peptide signals detected. The less abundant proteins are in Q1 and the most abundant ones in Q5.

With this classification of proteins according to their abundance, the percentage of acetylated proteins belonging to each quantile was calculated. This demonstrated the enrichment of acetylated proteins in high abundance proteins **(Fig. 3 B)**.

To calculate the percentage of the biological processes represented in the each quantile and acetylated proteins, all the proteins belonging to each quantile were analysed using PantherDB. Global proteome represents all the annotated ORF in *E. coli* genome. **Supplementary Figure 5**.

Complementation experiments. *E. coli* wild type strain and *cobB* mutant were transformed with the empty vector, p*BAD24,* or the pBAD24 carrying the native and mutant version of the *cobB* gene (mutation was its histidine 110 that forms part of its catalytic site). All transformants were grown overnight in 2 ml LB precultures supplemented with acetate 60 mM and ampicillin 100 µg/mL at 37ºC with orbital incubation. Precultures were cleaned three times with 0.9% NaCl and used for the inoculation of 25 mL minimal media with 60 mM acetate as sole carbon source. Growth of all transformants was followed for 24 hours (**Supp. Figure 6**)

Western blotting. Acs and AceA proteins were resolved in 12% acrylamide SDS page. Blotting was performed as into PVDF membranes. Membranes were proofed against rabbit anti acetyl lysine (InmuneChem, Burnaby, Canada) according to the manufacturer instructions. A goat anti rabbit antibody conjugated with HPR (Santa Cruz Biotechnology, Heidelberg, Germany) was used.

Deacetylation assays LC-MS.

Protein digestion. 20 µg of each sample were resuspended in lysis buffer containing 8 M urea, 50 mM ammonium bicarbonate and complete EDTA-free protease inhibitor Cocktail (Roche, reduced with 4 mM DTT for 25 min at 56°C, and alkylated with 8 mM iodoacetamide for 30 min at room temperature, in the dark). The proteins were first digested with LysC (1:50 ratio w/w) at 37°C for 4 h. Afterwards, samples were diluted 4-fold in 50 mM ammonium bicarbonate, and digested overnight with trypsin (1:50 ratio w/w) at 37°C.

Mass spectrometry: RP-nanoLC-MS/MS. Samples were diluted with 10% formic acid (FA) / 5% DMSO and 25 ng of the sample was analyzed using a Proxeon Easy-nLC100 (Thermo Scientific) connected to an Orbitrap Q-Exactive mass spectrometer. Samples were first trapped (Dr Maisch Reprosil C18, 3 um, 2 cm x 100 µm) before being separated on an analytical column (Agilent Poroshell EC-C18, 2.7 µm, 40 cm x 50 µm), using a gradient of 60 min at a column flow of 150 nl min^{-1}. Trapping was performed at 8 µL/min for 10 min in solvent A (0.1 M acetic acid in water) and the gradient was as follows: 7- 30% solvent B (0.1 M acetic acid in acetonitrile) in 31 min,

30-100% in 3 min, 100% solvent B for 5 min, and 7% solvent B for 13 min. Nanospray was performed at 1.7 kV using a fused silica capillary that was pulled in-house and coated with gold (o.d. 360 µm; i.d. 20 µm; tip i.d. 10 µm). The mass spectrometers were used in a data-dependent mode, which automatically switched between MS and MS/MS. Full scan MS spectra from *m/z* 350 – 1500 were acquired at a resolution of 35.000 at *m/z 400* after the accumulation to a target value of 3E6. Up to ten most intense precursor ions were selected for fragmentation. HCD fragmentation was performed at normalised collision energy of 25% after the accumulation to a target value of 5e4. MS2 was acquired at a resolution of 17.500 and dynamic exclusion was enabled (exclusion size list 500, exclusion duration 10 s).

LC-MS/MS data analysis. Raw data was analysed by MaxQuant (version 1.3.0.5) (Cox & Mann, 2008). Andromeda (Cox *et al*, 2011) was used to search the MS/MS data against the Uniprot *E.coli* MG1655 database (version v2012-09, 4431 sequences), including a list of common contaminants and concatenated with the reversed version of all sequences. Trypsin/P was chosen as cleavage specificity allowing three missed cleavages. Carbamidomethylation (C) was set as a fixed modification, while Oxidation (M), Acetyl (Protein N-term) and Acetyl (K) were used as variable modifications.

The database searches were performed using a peptide tolerance of 20 ppm for the first search and 6 ppm for the main search. HCD fragment ion tolerance was set to 20 ppm. Data filtering was carried out using the following parameters: Peptide False Discovery Rate (FDR) was set to 1%; max peptide PEP was set to 1; minimum peptide length was set to 5; minimum razor peptides were set to 1; peptides used for protein quantification was set to razor and unique peptides; the re-quantify option was enabled. The $\log_2$ intensities of the acetylated peptides were normalized subtracting the $\log_2$ median of all the peptide intensities in the same run.
