# Supplementary figures and images for: Protein acetylation affects acetate metabolism, motility and acid stress response in Escherichia coli
Source: Mol Syst Biol. 2014 Nov 28;10(11):762. doi: 10.15252/msb.20145227 (PMC4299603; doi:10.15252/msb.20145227)

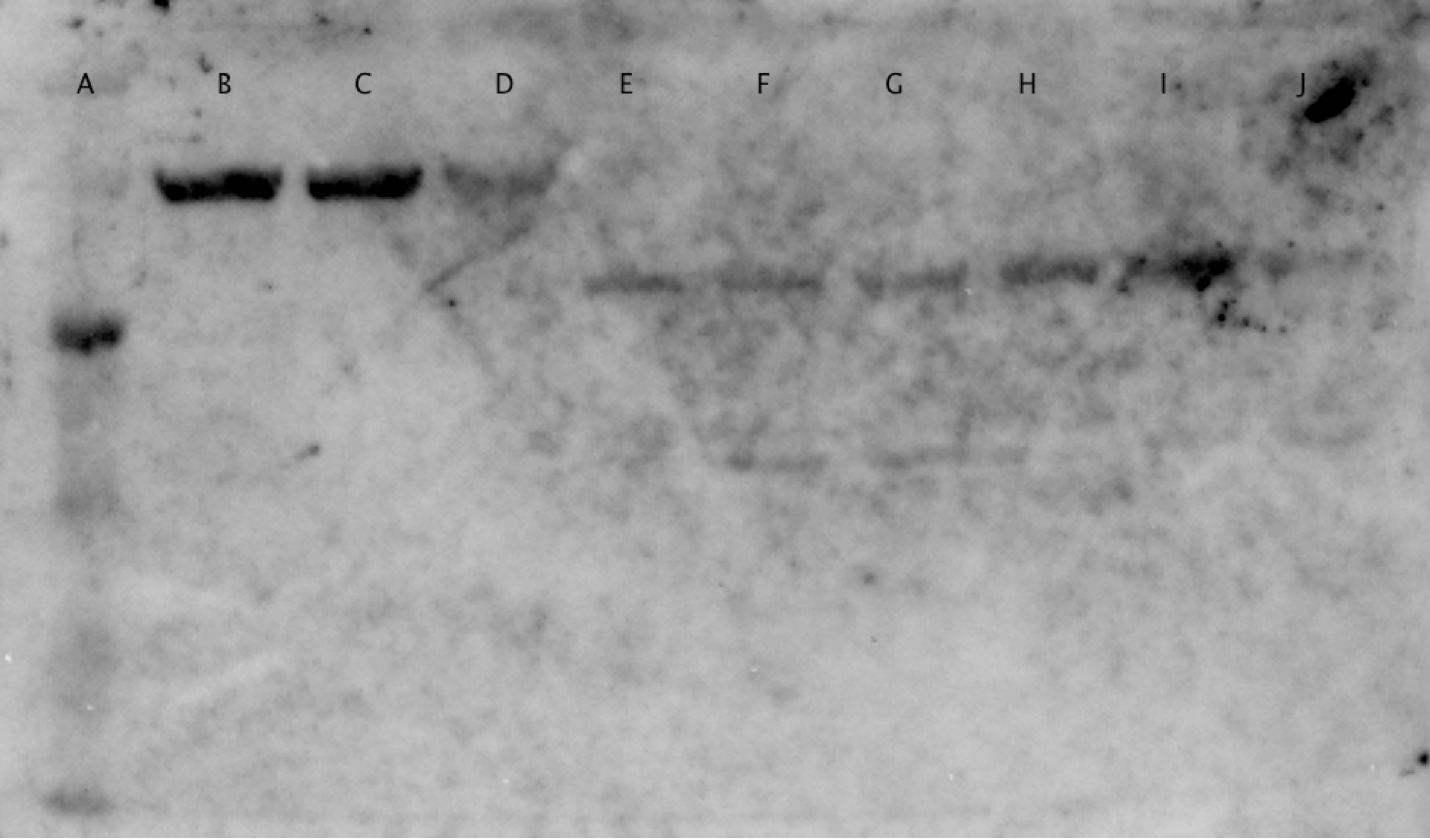

Supplement: Supplementary file 32 — Source Data for Figure 4 [file msb0010-0762-sd32.zip › Source Data Figure 4/Source Data Western Blots Figure 4.tif]
